# Interleukin 7 Plays a Role in T Lymphocyte Apoptosis Inhibition Driven by Mesenchymal Stem Cell without Favoring Proliferation and Cytokines Secretion

**DOI:** 10.1371/journal.pone.0106673

**Published:** 2014-09-03

**Authors:** Marilia Normanton, Heliene Alvarenga, Nelson Hamerschlak, Andreza Ribeiro, Andrea Kondo, Luiz Vicente Rizzo, Luciana Cavalheiro Marti

**Affiliations:** 1 Hospital Israelita Albert Einstein, Instituto Israelita de Ensino e Pesquisa Albert Einstein (IIEP-AE), São Paulo, SP, Brasil; 2 Hospital Israelita Albert Einstein, Bone Marrow Transplant Program, São Paulo, SP, Brasil; 3 Hospital Israelita Albert Einstein, Oncology Department, São Paulo, SP, Brasil; 4 Hospital Israelita Albert Einstein, Blood Bank Department, São Paulo, SP, Brasil; 5 Programa de Pós-graduação em Alergia e Imunopatologia, Faculdade de Medicina da Universidade de São Paulo (FMUSP), São Paulo, SP, Brasil; New York University, United States of America

## Abstract

Since 2004, when a case report describing the use of human mesenchymal stem cells (hMSCs) infusion as a therapy for GVHD after bone marrow transplantation, a new perspective in MSC function emerged. Since then hMSCs immunomodulatory potential became the target of several studies. Although great progress has been made in our understanding of hMSCs, their effect on T cell remains obscure. Our study has confirmed the already described effect of hMSCs on lymphocytes proliferation and survival. We also show that the impairment of lymphocyte proliferation and apoptosis is contact-independent and occurs in a prostaglandin-independent manner. A potential correlation between IL-7 and hMSCs effect is suggested, as we observed an increase in IL-7 receptors (CD127) on lymphocyte membrane in MSC presence. Additionally, blocking IL-7 in hMSCs-lymphocytes co-cultures increased lymphocytes apoptosis and we also have demonstrated that hMSCs are able to produce this interleukin. Moreover, we found that during Th1/Th17 differentiation *in vitro*, hMSCs presence leads to Th1/Th17 cells with reduced capacity of INF-y and IL-17 secretion respectively, regardless of having several pro-inflammatory cytokines in culture. We did not confirm an increment of Treg in these cultures, but a reduced percentage of INF-y/IL-17 secreting cells was observed, suggesting that the ratio between anti and pro-inflammatory cells changed. This changed ratio is very important to GvHD therapy and links hMSCs to an anti-inflammatory role. Taken together, our findings provide important preliminary results on the lymphocyte pathway modulated by MSCs and may contribute for developing novel treatments and therapeutic targets for GvHD and others autoimmune diseases.

## Introduction

The immunomodulatory activity of mesenchymal stem cells is at the basis of their therapeutic use in graft versus host disease (GVHD) which was first reported by Le Blanc et al. in 2004. After receiving human mesenchymal stem cells (hMSCs) infusions, the patient had significant clinical improvement without showing adverse reactions [1]. Since then, several studies have addressed the potential use of hMSCs to down regulate immune responses. Mesenchymal stem cells (MSCs), also known as mesenchymal stromal cells, are adult non-hematopoietic stem cells that were first described as plastic-adherent fibroblast-like cells capable of self-renewal and of differentiation into other cell type [2], [3].

In 2006, the International Society for Cellular Therapy proposed a set of phenotypic and functional criteria to define MSCs [4], which isolation is possible from a variety of sources [5]–[7]. However, the bone marrow still is the most studied source of hMSCs, mainly because it is the one used in cell therapy clinical trials. Although MSC immunomodulation occurs by distinct cell signaling pathways in humans and in animal models, an effector activity common to mouse, monkey and hMSCs is the inhibition of lymphocyte proliferation. Several causes of this inhibition have been described such as depletion of tryptophan by indoleamine-2,3-dioxigenase (IDO), up-regulation of prostaglandins secretion, oxidative stress or reactive oxygen species and nitrogen radicals (mainly in animal models) or, in humans, the action of IFN-γ [8], [9]. The proliferative responses of T cells, whether stimulated by alloantigen [10]–[12], antigenic peptides [13], mitogens [10], [14] or by anti-CD3/CD28 antibodies, were suppressed in the presence of MSCs suggesting non-specificity of mechanisms.

However, as bone marrow derived MSCs reside in hematopoietic stem cell niches they primarily affect the differentiation and maintenance of hematopoietic stem cells. Most likely MSCs influence the survival of their neighbor cells, either by contact-dependent interactions or by secreting soluble molecules [15], [16]. Recently it was shown that MSCs secrete multiple cytokines, promote angiogenesis and have distinct effects on chemotaxis and apoptosis [17].

Among the cytokines that affect T cell proliferation, interleukin-7 (IL-7), is a cytokine secreted by stromal cells that is critical for the development, homeostatic proliferation and survival of naïve T cells. The absence of IL7 or any of its proximal signaling components leads to a severe combined immunodeficiency (SCID). IL-7 is detected by a two-part receptor on lymphocytes, consisting by a γ-chain that is shared by multiple cytokines and a more specific receptor IL-7Rα. IL-7R signals through the Jak/STAT and PI3K/Akt signaling pathways, both of which are known to have effects on cell survival, growth and metabolism. Although, the specific role and mechanism by which IL-7R may influence each of these processes *in vivo*, has not been fully determined [18]–[20].

Given that relatively little is known on the mechanisms that underlie the control of GVHD and T cell proliferation by MSCs in humans, we undertook the present investigation. To this end we studied the effect of hMSCs on phytohemagglutinin-stimulated lymphocytes cultures. Phytohemagglutinin (PHA) is a mitogen that stimulates very robust activation and proliferation of T cells. We evaluated the cytokine secretion patterns by lymphocytes when in contact with MSCs in culture, their effect on activation-induced lymphocyte cell death (AICD) rates, and the participation of prostaglandins and IL-7 in the death-signaling pathway. Because Th1/Th-17 and INF-γ/IL-17 participate in GVHD-inflammation [21] and given the beneficial effect of hMSCs infusion at reducing the intensity of GVHD, we also investigated whether MSCs would influence Th1 and Th17 differentiation or INF-γ and IL-17 secretion.

Briefly, we found that hMSCs suppression of lymphocyte proliferation and apoptosis did not depend on cell-contact and was not mediated by prostaglandins. In addition, depletion of IL-7 in co-cultures increased apoptosis suggesting that T cell survival is promoted by this cytokine. Furthermore, hMSCs reduced INF-γ and IL-17 synthesis and the frequency of Th1/Th17 cells in co-culture with no increment of Treg cells. Our findings pave the way to a better understanding on how hMSCs act on lymphocytes and may contribute for further applications of these powerful therapeutic cells.

## Materials and Methods

### Cells isolation

hMSCs were obtained from health bone marrow donors, by washing the cells trapped in the dischargeable collection filter as previously described by Deus et al [22]. The lymphocytes were obtained from health donor’s peripheral blood following the signing of informed consent (Approved by Hospital Albert Einstein Ethical Committee -10/1412; CAAE: 06592712.4.0000.0071).

Peripheral blood cells were diluted 1∶3 (vol/vol) with Phosphate Buffered Saline (PBS). Next, the suspension were transferred to a 50 mL conical tube containing 20 mL of Ficoll-Paque 1.077 density (GE Healthcare, United Kingdom) and centrifuged (30 min/500 g) at 22°C. Then, the cells from the interface were collected, ressuspended and centrifuged again (5 min/500 g). The supernatant was discarded and cells ressuspended with DMEM-LG, supplemented with 10% FBS, 1% antibiotic-antimycotic and 2 mM L-glutamine, in order to achieve 1×10^6^ cells/mL. In order to deplete monocytes the cells were placed in a petri dish previously coated with human albumin 20% (Sandoz, Germany), next the cells were incubated for 1 hour in humidified 5% CO2 incubators at 37°C, the non-adherent cells were removed, and the efficiency of depletion was verified by flow cytometry. The mentioned culture media, supplements and PBS were bought from Gibco (Carlsbad, CA).

Bone marrow collection filters were washed with 20 mL DMEM-LG; the cells were diluted 1∶3 (vol/vol) with Phosphate Buffered Saline (PBS). Next, the suspension were transferred to a 50 mL conical tube containing 20 mL of Ficoll-Paque 1.077 density (GE Healthcare, United Kingdom) and centrifuged (30 min/500 g) at 22°C. Then, the cells from the interface were collected, ressuspended and centrifuged again (5 min/500 g). The supernatant was discarded and cells ressuspended with DMEM-LG, supplemented with 10% FBS, 1% antibiotic-antimycotic, 2 mM L-glutamine, in order to achieve 1×10^5^ cells/mL. The mentioned culture media, supplements and PBS were bought from Gibco (Carlsbad, CA). Next, cells (5 mL) were cultivated into 25 cm^2^ flasks for 48 hours and maintained in humidified 5% CO_2_ incubators at 37°C to favor the attachment of the hMSCs to the flask bottom. The non-adherent cells were removed, the adherent layer was washed twice with DMEM-LG, than maintained in culture until the 4^th^ passage.

hMSCs were characterized by flow cytometry and checked for the ability to differentiate in osteocytes, chondrocytes or adipocytes as previously published by our group and briefly described below [22].

### Mesenchymal stem cell characterization by flow cytometric expression markers

Cells from passage four were used to analysis of cell surface markers. The cells were washed with PBS, and then detached from the plastic with TryPLE (Gibco Carlsbad, CA). Next, the cells were stained for CD106-FITC (clone: 51-10C9), CD73-PE (clone: AD2), CD34-PE (clone: My10), CD105-PE-CF594 (clone: 266), CD90-PE-Cy7 (clone: SE10), CD29-APC (clone: MAR04), CD14-Alexa 700 (clone: M5E2) from BD Pharmingen (San Diego – CA), CD44-PerCPCy5 (clone: G44-26), HLA-DR-APC-H7 (clone: G46-6) from Biosciences (San Jose – CA), CD45-V500 (clone: H130) and CD31-V450 (clone: WM59) from Biolegend (San Diego – CA), and for fluorescence minus one (FMO). After staining, the tubes were incubated at room temperature for 30 minutes, followed by a wash step; the cell pellet was ressuspended and measurements were performed using FACSARIA equipment (BD Biosciences).

The human mesenchymal stem cells (hMSCs) used in our assays fulfilled this identity criteria established by the International Society for Cell Therapy (ISCT), as shown by Figure S1.

### Mesenchymal stem cell characterization by adipocyte, chondrocyte and osteocyte differentiation

After the establishment of hMSCs cultures on the fourth passage, the cells were differentiated into adipocytes, osteoblasts, and chondrocytes.

Adipogenesis was induced by addition of an adipogenic medium to hMSCs culture, this medium was comprised by Alpha-MEM supplemented with 10% FBS, 1 µm dexamethasone, 100 µg/mL 3-Isobutyl-1-methylxanthine, 10 µg/mL insulin and 100 µM indomethacin. The adipogenic medium was changed every other day for 3 weeks. After 3 weeks in culture the cells were stained to evidence lipid droplets formation. The cells were fixed in 4% paraformaldehyde for 30 minutes, washed, dehydrated in 60% isopropanol for 2 to 5 minutes, and stained with 0.5% Oil Red O (O-0625) in 100% isopropanol previously diluted in water.

Osteogenesis was induced by addition of an osteogenic medium which was comprised by Alpha-MEM supplemented with 10% FBS, 1 µm dexamethasone, 2 µg/mL ascorbic acid and 10 µm β-glycerophosphate. The osteogenic medium was changed every other day for 3 weeks. After 3 weeks in culture the cells were stained with Alizarin Red to evidence the calcium deposition. Next, the cells were fixed in 4% paraformaldehyde for 30 minutes, washed with distilled water, stained with Alizarin Red (2 g in 100 mL of distillated water) pH 4.2 (A5533) for 5 to 10 minutes and thoroughly washed.

Chondrogenesis was induced by addition of a chondrogenic medium which was comprised by Alpha-MEM supplemented with 10% FBS, 1 µm dexamethasone, 2 ug/mL ascorbic acid, 6,25 ug/mL insulin, and 10 ng/mL TGF-β. Chondrogenic medium was changed every other day for 3 weeks. After 3 weeks in culture the cells were stained with toluidine blue for evidence the proteoglycans enriched matrix. For toluidine blue, the cells were fixed with ethanol 70% for 1 minute, ethanol 90% for 1 minute and absolute ethanol for 1 minute, then toluidine blue was added (1 g toluidine blue, 1 g sodium borate/100 mL of water) (198161).

The mentioned culture media and FBS were bought from Gibco (Carlsbad, CA) and the other reagents were all from Sigma (St Louis, MO).

The human mesenchymal stem cells (hMSCs) used in our assays fulfilled this identity criteria established by the International Society for Cell Therapy (ISCT), as shown by Figure S2.

### Cell culture

hMSCs at 4^th^ passage were transferred to a 6-well plate and maintained in culture until reaching 80% confluence. The lymphocytes were suspended in Xvivo15 (Cambrex, Walkersville, MD), supplemented with 1% human pooled serum AB (Life Technologies, Carlsbad, CA) and 1% antibiotic-antimycotic (Gibco, Carlsbad, CA) then stimulated with 1 µg of phytohemagglutinin (PHA)/1×10^6^ lymphocytes. Approximately 1×10^6^ lymphocytes were added to 1×10^5^ hMSCs. The co-cultures were incubated for 24, 48 and/or 72 hours before assessing for lymphocyte apoptosis/proliferation. Lymphocytes were also co-cultivated with hMSCs and PHA separated by transwell 0.2 µm - anopore membrane (Nunc Kamstrup, Denamark). Control cultures of lymphocytes without PHA stimulation were also included in all experiments.

### Mixed leucocyte reaction (MLR)

Dendritic cells were derived from monocytes selected for CD14 by magnetic selection column (Myltenyi Biotec, Bergisch Gladbach, Germany). The CD14^+^ cell population was dispensed into six-well plates containing X-vivo 15 medium (Cambrex) supplemented with antibiotic-antimycotics (Gibco). To generate immature dendritic cells (DCs), the cells were cultured in the presence of 20 ng/mL recombinant IL-4 (rIL-4) and 50 ng/ml rGM-CSF (both from R&D Systems, Minneapolis, MN) for six days (indicated as D6 cells). Mature DCs were obtained after 24 h (D7) after stimulation of the immature DC culture with 10 ng/mL rTNF-α (R&D System) plus 0.01 mmol/L of PGE2 (Sigma) [23].

In order to set up the MLR, matured DCs were irradiated at 1,500 rads and co-cultured with allogeneic lymphocytes at the concentration of 1 DC:100 Lymphocytes (1×10^4^:1×10^6^ respectively) in presence or absence of 1×10^5^ allogeneic hMSCs. The proliferation rates were determined after four days of co-culture. Lymphocyte proliferation was measured by KI-67 nuclear staining on CD3+ gated population. The lymphocytes were gated based on SSC vs FSC, followed by CD3 gating; the selected events were analyzed in a second plot for KI-67 expression. The proliferation negative control was lymphocytes without allogeneic DCs stimulation.

### Treatment of cultures with Indomethacin, interleukin 7 or anti-IL-7 antibody

Co-cultures of PHA-stimulated lymphocytes and hMSCs were treated with different concentrations of Indomethacin (5, 25, 50 and 100 ng/mL) in order to verify if the blocking of prostaglandin synthesis would interfere with the anti-proliferative effects of hMSCs. Co-cultures of PHA-stimulated lymphocytes and hMSCs were also treated with different concentrations of polyclonal neutralizing goat anti-human IL-7 antibody (10 and 20 ul) (R&D Systems) or with rIL-7 (10 and 20 ng/mL, R&D systems), in order to verify a possible activity of this cytokine on the anti-apoptotic effect of hMSCs on lymphocytes.

### Th1 and Th17 differentiation

Naive T lymphocytes derived from healthy volunteers peripheral blood, (CD4, CD45RA), were separated by indirect cell targeting by using Naive CD4^+^ T Cell Isolation Kit II (Miltenyi biotec) according manufactures instruction. Naïve T cells (80% purity) were stimulated with CD3/CD28 microspheres (Life Technologies), and cultured for 5 days, treated with 10 ng/mL of IL1β, IL6, IL23 and 5 ng/mL of TGFβ (R&D Systems) for Th1 and with monoclonal antibodies (mAb) anti-IFN-y (Clone: 25723.11) and anti-IL2 (Clone: 5344.111) at 1∶50 dilution bought from BD Biosciences for Th17. This protocol of naïve T cells to Th1/Th17 differentiation was carried out in presence or absence of hMSCs.

### T-cell apoptosis and proliferation assay

Apoptosis and proliferation were measured by flow cytometric assays. Lymphocytes were stained according to manufactures instructions, using two different combinations: First combination was Annexin V-FITC, propidium iodide-PI and CD3-APC (clone: HIT3a) from BD Pharmingen, (CA, San Diego). The second combination used was KI-67-FTIC (clone: MOPC-21), Caspase-3 activated-PE (clone: C92-605), CD4-APC (clone: RPA-T4) from BD Pharmingen and CD3-Per CP Cy5.5 (clone: SK7) from BD Biosciences. The corresponding isotype controls were used as control for both staining protocols.

Briefly, for testing apoptosis the lymphocytes were ressuspendend with Annexin-V binding buffer (BD Biosciences) and stained for Annexin V, PI and CD3 for 15 minutes; the samples were acquired for flow cytometric analysis within 30 minutes after finishing incubation. For proliferation and apoptosis, staining for surface markers as CD3 and CD4 was performed and the cells were incubated for 30 minutes following fixation using BD FACS Lysing solution (BD Biosciences). Next, the cells were permeabilized using BD FACS Permeabilization solution 2 (BD Biosciences) and the intracellular staining was performed. After washing, the cell suspension was submitted to FACS analysis.

### T-cell BrdU proliferation assay

Lymphocytes were stimulated with 1 µg of phytohemagglutinin (PHA)/1×10^6^ lymphocytes. Approximately 1×10^6^ lymphocytes were added to 1×10^5^ hMSCs or cultured alone. These cultures were incubated for 48, and then pulsed with 10 µM of BrdU (BD Pharmingen)/1×10^6^ lymphocytes for another 24 hours. The lymphocytes were first stained for surface antigens such as CD3-PerCP Cy5.5, CD4-PE, then fixed and permeabilized. Next the cells were treated for 1 hour with DNase in order to expose the incorporated BrdU. Next, the staining with anti-BrdU-FITC was performed following manufactures’ instructions of FITC BrdU flow kit (BD Pharmingen).

Lymphocyte proliferation was measured by BrdU-FITC staining on CD3+ gated population. The proliferation negative controls were lymphocytes without PHA stimulation.

We also have used BD Trucount tubes (BD Biosciences) to determine the absolute number of proliferating T lymphocyte. The cells absolute number was calculated by using the following formula:
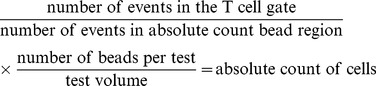



### Intracellular IL-7 detection on mesenchymal stem cells

To determine whether hMSCs produce IL-7 when in co-cultivation with lymphocytes, the cultures were maintained for 24 hours followed by BFA addition for 4 hours. hMSCs were stained with anti-CD105-PE (clone: 43A3; Biolegend – San Diego - CA). After 30 minutes of incubation the cells were washed, next fixed with lyses buffer (lyses buffer – BD Biosciences, San Diego, CA) followed by permeabilization (permeabilization solution 2– BD Biosciences). Next, we proceed the intracellular staining with polyclonal goat anti-human IL-7 antibody, followed by the secondary anti-goat FITC, the same were performed using isotype control.

### Dendritic cells characterization

Dendritic cells were characterized by flow cytometry regarding there phenotype and maturation status according to the intensity of CD14, CD209, CD80, CD83 and CD86 expression as showed in Figure S3. The staining of the cells was performed using commercially available monoclonal antibodies (mAbs) according to the manufacturer’s instructions. Briefly, the cells were stained for surface markers with the selected mAbs and incubated in the dark for 30 min at room temperature, followed by flow cytometry acquisition. The mAbs used to evaluate DC and its maturation were as follows: CD14-FITC (clone: M5E2), CD80-PE (clone: L307.4), CD83-PE (clone: HB15e), CD86-PE (clone: 2331), and CD209-PE (clone: DCN46) all purchased from BD Pharmingen, HLA-DR-PerCP-Cy5.5 (clone: L243) from BD Biosciences and corresponding isotype control.

### Th17 cell and regulatory T cell characterization

T cells in co-culture were characterized by flow cytometry assay and stained according to manufactures instruction. Briefly, for Th17 cells, the staining for surface markers CD4-APC (clone: RPA-T4), CD8-PE (clone: RPA-T8), CD45RA-FITC (clone: HI100), CD45RO-PE (clone: UCHL1), CD3-APC (clone: HIT3a) or CD3-Per CP-Cy5.5 (clone: SK7) from BD Pharmingen, CD4-APC-Cy7 (clone: SK3) and CD45 Per CP-Cy5.5 (clone: 2D1) from BD Biosciences, CCR5-FITC (clone: 45502) and CCR6-PE (clone: 53103) from R&D System, was performed and cells were incubated for 30 minutes following fixation using BD FACS Lysing solution (BD Biosciences). Next, cells were permeabilized using BD FACS Permeabilization solution II (BD Biosciences) and the intracellular staining was performed with Tbet-Per CP-Cy5.5 (clone: 4-46), anti-IL17A-FITC (clone: N49-653) from BD Pharmingen, RORγt-PE (clone: AFKJS-9) from eBiosciences, IFN-γ-FITC (clone: 25723.11) from BD Biosciences and the corresponding isotype control.

For Treg cells, staining for surface markers CD45-PE-Cy7 (clone: HI-30), CD3-APC-Cy7 (clone: SK-7), CD4-FITC (clone: RPA-T4), CD25-APC (Clone: M-A251) and CD127-PercP-Cy5.5 (clone: HIL-7R-M21), was performed and cells were incubated for 30 minutes following fixation/permeabilization using Foxp3 staining Kit (BD Biosciences, San Jose, CA) and intracellular staining FoxP3-PE (clone: 259D/C7). FMO control was performed for CD25-APC and Foxp3-PE.

### T-cell surface expression of CD127

CD127 was measured by flow cytometric assays. Lymphocytes were stained according to manufacture instructions for CD3-FITC (clone: HIT3a), CD127-PercP-Cy5.5 (clone: HIL-7R-M21), CD4-APC (clone: RPA-T4) from BD Pharmingen and corresponding isotype controls. Briefly, the staining for surface markers CD3, CD4 and CD127 was performed and cells were incubated for 30 minutes followed by fixation using BD FACS Lysing solution BD Biosciences.

### FACS analysis

Analysis of stained cells was performed using the BD FACSARIA equipment (BD Biosciences) and at least 10,000 events were acquired. The results were analyzed by FACSDIVA and/or FlowJo software (Treestar, Eugene, OR).

### Statistical Analysis

GraphPad Prisma Program was used to perform the statistical analyses. Statistical significance was calculated by Student’s *t*-test or ANOVA with Bonferroni correction for multiple comparisons; *p*<0.05 was considered significant.

## Results

### Mesenchymal stem cell reduces the proliferation of PHA and allogeneic DC stimulated T lymphocytes, and this suppression is not contact-dependent

**Figure 1:**
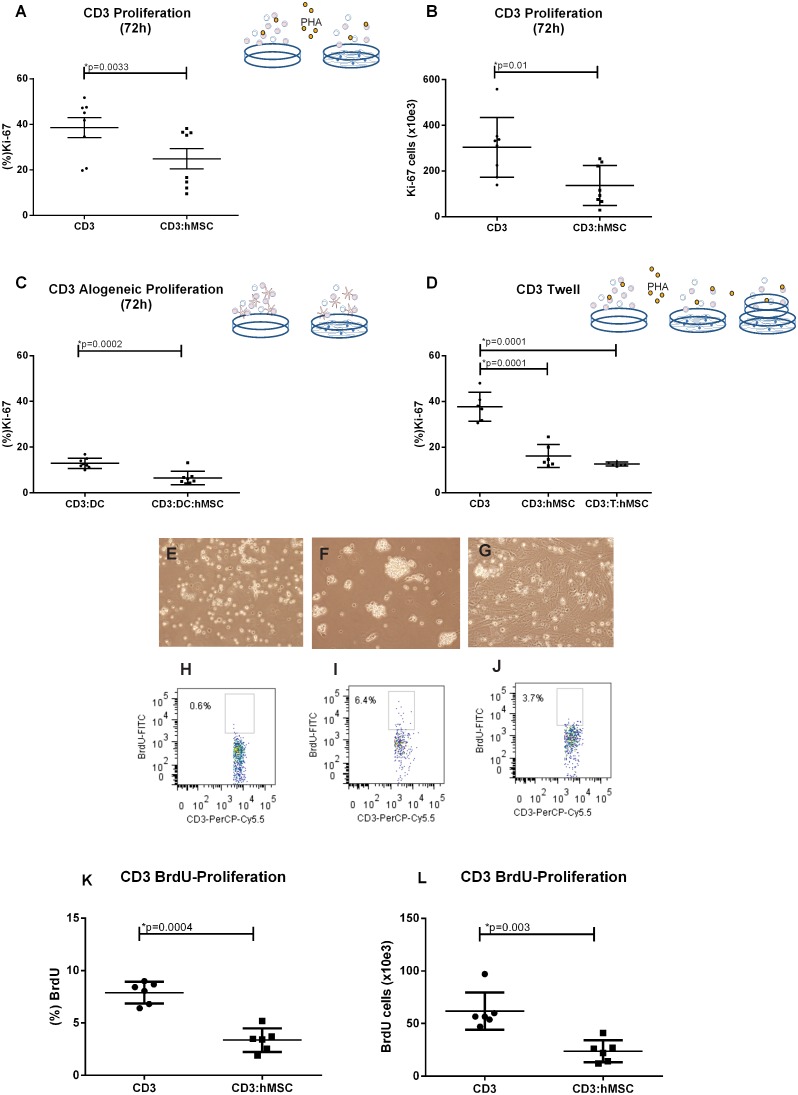
hMSCs reduces proliferation of stimulated lymphocytes in a contact-independent manner. (A–B) A significant proliferation reduction of PHA stimulated lymphocytes (CD3) (38.58±12.39%) assessed by expression of KI-67 was observed in presence of hMSCs (CD3: hMSC) (24.39±12.39%), the same was observed when absolute number KI-67 expressing cells was evaluated decreasing from (3.0×10^5^±1.3×10^5^) up to (1.7×10^5^±0.9×10^5^) (n = 8). (C) A significant proliferation reduction of DC stimulated lymphocytes (CD3: DC) (12.95±2.23%) assessed by expression of KI-67 was also observed in presence of hMSCs (CD3:DC:hMSC) (6.5±2.23%). (n = 8). (D) There was significant reduction of lymphocytes proliferation assessed by expression of KI-67, when in presence of hMSCs (CD3: hMSC) (16.18±4.99%) and with transwell (CD3:T:hMSC) (12.67±0.83%) if compared to the control (CD3) (37.67±6.63) (n = 6). (E–G) Figures showing the cells after 72 hs of culture (E) Lymphocytes without stimulation (F) Lymphocytes PHA stimulated (G) Lymphocytes PHA stimulated in co-cultures with hMSCs (H–J) Figures showing the (%) of BrdU (H) Lymphocytes without stimulation (I) Lymphocytes PHA stimulated (J) Lymphocytes PHA stimulated in co-culture with hMSCs (K) A significant proliferation reduction of PHA stimulated lymphocytes (CD3) (7.89±1.05%) assessed by BrdU incorporation was observed in presence of hMSCs (CD3:hMSC) (3.37±1.12%), (L) the same was observed when absolute number of BrdU expressing cells were evaluated in absence (0.6×10^5^±0.2×10^5^) or presence of hMSCs (0.2×10^5^±0.1×10^5^) (n = 6). Significant p-values showed in the graphic.

CD3+ T cells PHA-stimulated and co-cultivated with hMSCs show 15% less cells expressing KI-67 compared with similarly stimulated cultures without hMSCs (Figure 1A), in absolute numbers, the cells expressing KI-67 decreased almost 50% from (3.0×10^5^±1.3×10^5^) up to (1.7×10^5^±0.9×10^5^) (Figure 1B). Suppression of T cell proliferation by hMSCs (of the order of 50%) was also observed in allo DC-stimulated lymphocyte cultures (Figure 1C) compared to allogeneic cultures in the absence of hMSCs, showing that hMSCs play a role in T lymphocytes clonal expansion, either to a specific or nonspecific stimulus response. The flow cytometry approach and data that originated the figures are exemplified in Figure S4.

hMSCs were maintained separated from lymphocytes in transwell cultures or allowed direct contact with lymphocytes in culture wells. PHA-stimulated proliferation was significantly reduced in both culture settings showing that the suppressive effect on lymphocytes exerted by hMSCs did not require contact between these cell populations (Figure 1D).

To confirm that the cells have not only a low cycling percentage by lower expression of KI-67 but also a reduced number of division in hMSCs presence, we also performed a proliferation assay by other technique, measuring BrdU incorporation, and we found that a reduction on T cell division by hMSCs presence (of the order of 50%) was also observed in both percentage and absolute number of cells (Figures 1E–L).

### Addition of Indomethacin to the cultures decrease PHA-stimulated T cell proliferation but in contrast tryptophan addition restores lymphocytes proliferation

**Figure 2:**
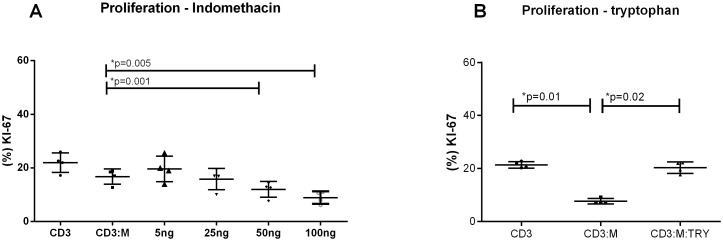
Indomethacin reduces PHA stimulated lymphocytes proliferation in presence of hMSCs and tryptophan addition to these cultures recovery the lymphocytes proliferation. (A) Proliferation control (CD3) (21.35±3.62%) is enhanced if compared to stimulated lymphocytes co-cutivated with hMSCs (CD3:M) (16.75±2.81). In addition, we observed that CD3:hMSC have their proliferation even more reduced when added of 50 ng (11.99±2.92%), or 100 ng (8.92±2.32%) of indomethacin. (B) Proliferation control (CD3) (21.43±3.38%) is enhanced if compared with stimulated lymphocytes co-cutivated with hMSCs (CD3:M) (7.55±1.66). However, tryptophan addition to hMSCs condition (CD3:M:TRY) recovered the proliferation (20.33±5.03) (n = 4). Significant p-values showed in the graphic.

Because prostaglandins and specially PGE2 are known suppressor molecules of lymphocyte proliferation it was of interest to check whether they participated in the suppression exerted by hMSCs on lymphocyte proliferation. However, the addition of indomethacin that blocks the cyclooxygenase 1 and 2, failed to abrogate the hMSCs-mediated suppression in PHA-stimulated lymphocytes. On the contrary, indomethacin treatment decreased in a dose-dependent manner the proliferation of lymphocytes (Figure 2A). The depletion of tryptophan by the stimulated activity of the enzyme indoleamine 2 3-dioxygenase is a common cause of decreased lymphocyte proliferation, because this aminoacid is essential for cellular proliferation. As shown in Figure 2B, the addition of tryptophan completely restored PHA-stimulated proliferation in hMSCs-lymphocytes co-cultures to the levels observed in cultures of lymphocytes lacking hMSCs. Therefore, we suggest that tryptophan depletion determined by hMSCs is an important component of their suppressive effect on lymphocytes.

### The rates of apoptosis/necrosis of PHA and allogeneic-DC-stimulated T lymphocytes are lower in co-culture with hMSCs and are partially contact-dependent

**Figure 3:**
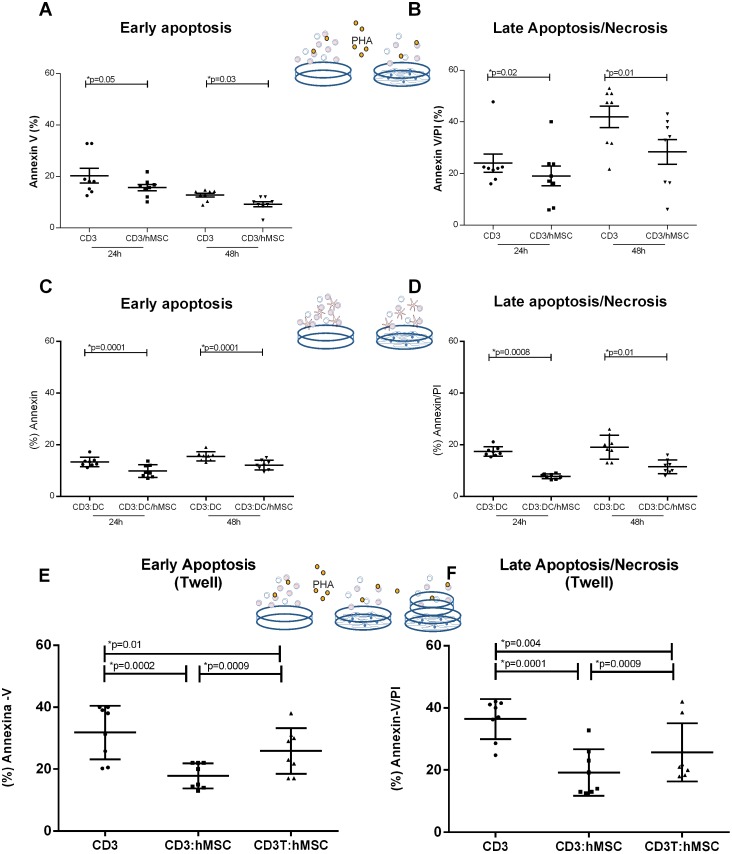
hMSCs presence reduces apoptosis and/or necrosis of stimulated lymphocytes and it is partially-contact dependent. (A) Early apoptosis in PHA stimulated lymphocytes co-cultivated (CD3/hMSC) or not (CD3) with hMSCs. There is a significant reduction after 24 and 48 hs of cultivation in hMSCs presence (15.61±3.55; 9.17±2.85%) as compared to hMSCs absence (20.28±8.03; 12.71±2.0%) respectively. (B) Late apoptosis/necrosis in PHA lymphocytes stimulated in presence (CD3/hMSC) or not (CD3) of hMSCs. There is a significant reduction after 24 and 48 hs of cultivation in hMSCs presence (19.05±10.83; 28.38±13.47%) as compared to hMSCs absence (24.00±9.91; 41.95±11.73%) respectively. (C) Early apoptosis in DC stimulated lymphocytes co-cultivated (CD3:DC/hMSC) or not (CD3:DC) with hMSCs. There is a significant reduction after 24 and 48 hs of cultivation in hMSCs presence (9.80±2.42; 12.09±1.87%) as compared to hMSCs absence (13.30±1.82; 15.49±21.82%) respectively. (D) Late apoptosis/necrosis in DC stimulated lymphocytes co-cultivated (CD3:DC/hMSC) or not (CD3:DC) with hMSCs. There is a significant reduction after 24 and 48 hs of cultivation in hMSCs presence (7.79±0.93; 11.46±2.64%) as compared to hMSCs absence (17.38±1.83; 19.09±4.64%) respectively. (E) and (F) show early apoptosis and the late apoptosis, respectively of stimulated lymphocytes (CD3/PHA) and co-cultivated with hMSCs (CD3/hMSC) using a 0.2 um transwell (CD3T/hMSC) for 72 hours. (E) There is a significant reduction of early apoptosis in presence of hMSCs (17.08±4.05%) if compared to control (31.84±8.65%) and in presence of transwell (25.85±7.39%). (F) A significant reduction of late apoptosis/necrosis of stimulated lymphocytes (36,41+6.42) is observed if compared to values obtained in presence of hMSCs (19.19±7.51%) and transwell (25.73±9.34%). (n = 8) Significant p-values showed in the graphic.

The reduced T lymphocyte proliferation rates observed in culture with hMSCs could reflect increased apoptosis of the former cells. However, co-cultures of hMSCs and CD3+ T cells stimulated with either PHA or allogeneic DC presented fewer apoptotic cells (Annexin V+) at 24 h of culture and also fewer apoptotic/necrotic cells (Annexin V+/Propidium Iodide +) at 48 h of culture in comparison with cultures that did not receive hMSCs (Figure 3A–D). The flow cytometry approach and data that originated the data in Figure 3 are exemplified in Figure S5. The results indicate that apoptosis or necrosis induction is not the cause of reduced proliferation by T cells when in co-culture with hMSCs. To evaluate whether the apoptosis-protective effect of hMSCs on co-cultured lymphocytes required contact between these two cell types, they were cultured separated by a transwell membrane or in conventional culture conditions that allow direct contact between the cells. The experiments showed that even separated by a membrane, hMSCs led to a reduction of lymphocytes apoptosis/necrosis, although the strongest protective effect occurred when cells were in contact (Figure 3E–F). The experiment suggests that soluble factors secreted by hMSCs also contribute to the reduction of cell death rates of lymphocytes.

### Interleukin 7 produced by hMSCs participates in the hMSCs-driven T Lymphocytes survival

**Figure 4:**
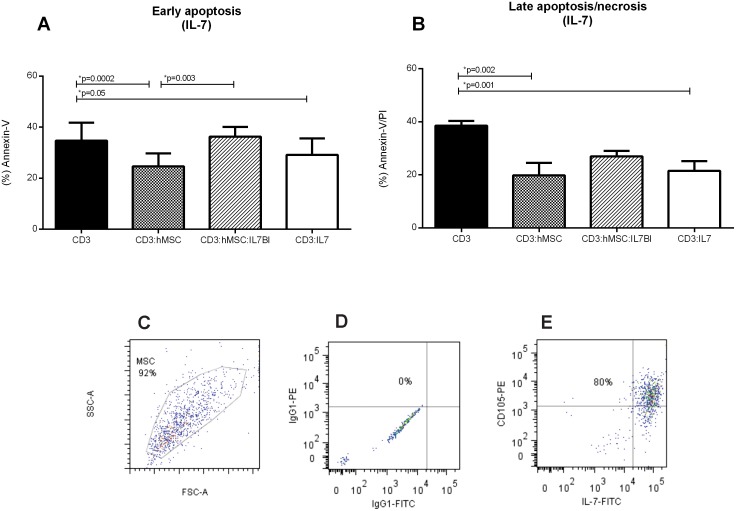
hMSCs produces Interleukin-7 which effect in PHA stimulated lymphocytes is likely hMSCs presence in early and late apoptosis. (A) Early apoptosis in PHA stimulated lymphocytes (CD3) is 34.7±7.05% and in presence of hMSC is reduced (CD3:hMSC) (24.6±5.11%) or stimulated lymphocytes plus interleukin-7 (CD:3IL-7) (29.12±6.4), but no difference is observed if compared with hMSCs co-cultivated cells plus IL-7 block agent (CD3:hMSC:IL-7Bl) (36.27±3.8%). (B) Late apoptosis/necrosis in PHA stimulated lymphocytes (CD3) is 38.55±1.76% and in presence of hMSCs is reduced (CD3:hMSC) (19.85±4.72%), in presence of IL-7 block agent (CD3:hMSC:IL-7Bl) (26.97±2.12%) or when stimulated lymphocytes plus interleukin-7 (CD3:IL-7) (21.52±3.65%). (C) The first plot show the gate on hMSCs by SSC vs FSC (D) the hMSCs gated cells are analyzed for isotype controls (E) the hMSCs gated cells are analyzed for CD105 and IL-7, we observed that 80% of the cells expressed CD105 and co-expressed IL-7. (n = 4) Significant p-values are showed in the graphic.

As IL-7 has been described as cytokine that promotes cell survival, it is a candidate among the apoptosis-protective soluble factors produced by hMSCs in co-culture with T lymphocytes. Blocking IL-7 by adding anti-IL-7 antibody to hMSCs/lymphocytes co-cultures significantly enhanced early apoptosis (Figure 4A) while the addition of recombinant IL-7 to the cultures further decreased apoptosis (Figure 4B). These results suggest that IL-7 participates in the hMSCs-driven reduction of apoptosis observed in co-cultured lymphocytes. That hMSCs do indeed produce IL-7 was shown by the intracellular staining of IL-7 in 80% of hMSCs as detected by cytometry (Figure 4C–E). In addition we found that the presence of hMSCs in the cultures moderately enhanced (from 37±10% to 44±10%) the frequency of T lymphocytes expressing the IL-7 receptor, CD127 (data not shown).

### The presence of hMSCs in Th1 or Th17 lymphocyte differentiation cultures results in lower frequencies of cells and modifies the relative frequency of T regulatory and IFN-γ/IL-17- secreting cells

**Figure 5:**
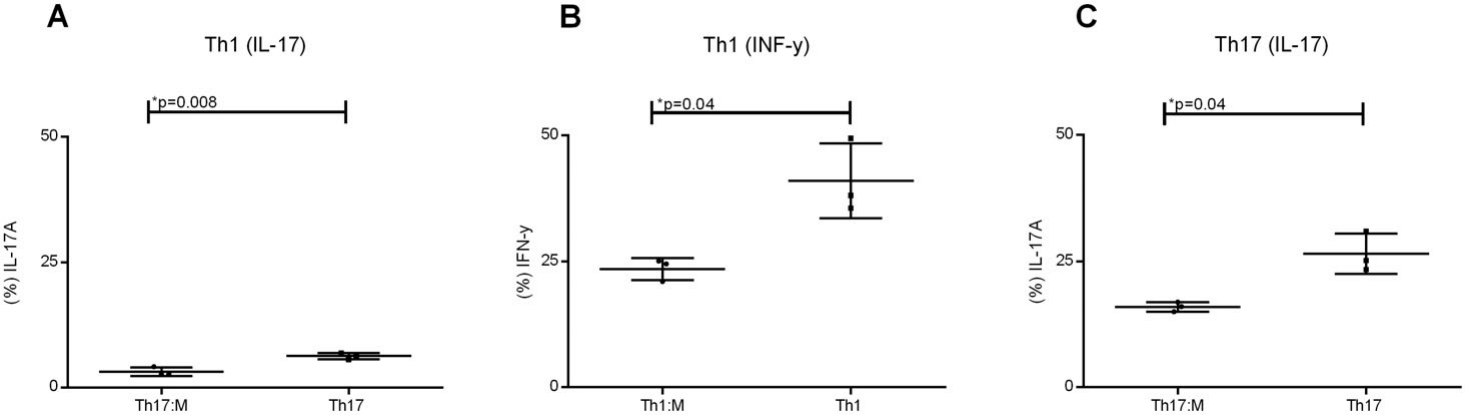
Naïve T cells differentiated into Th1 and Th17 in presence of hMSCs secrete approximately 50% less INF-γ and IL-17. (A) Naïve T cells differentiated for Th1 in presence of hMSCs secrete less IL-17 (3.17±0.86%) than the ones differentiated in their presence (6.25±0.63%) (B) Naïve T cells differentiated for Th1 in presence of hMSCs secrete less INF-y (23.53±2.21%) than the ones differentiated in their presence (40.97±7.41%) (C) Naïve T cells differentiated for Th17 in presence of hMSCs secrete less IL-17 (15.97±0.95%) than the ones differentiated in their presence (26.53±3.97) (n = 3). Significant p-values showed in the graphic.

Since IFN-**γ** and IL-17 have been reported to be the main cytokines in the development and maintenance of GvHD, we also investigated whether the presence of hMSCs during *in vitro* differentiation of naïve T cells to Th1 or Th17 would affect the amount of cytokine production by these cells. As shown in Figure 5, the frequencies of IL-17- or IFN-γ expressing T cells that were differentiated by Th1-promoting protocols in the presence of hMSCs were about 50% lower than in the controls without hMSCs. The frequency of IL-17–expressing cells in cultures that underwent the Th17 differentiation protocol in the presence of hMSCs were also 40% lower than in control cultures not exposed to hMSCs. The FACS data are supplied in Figure S6.

**Figure 6:**
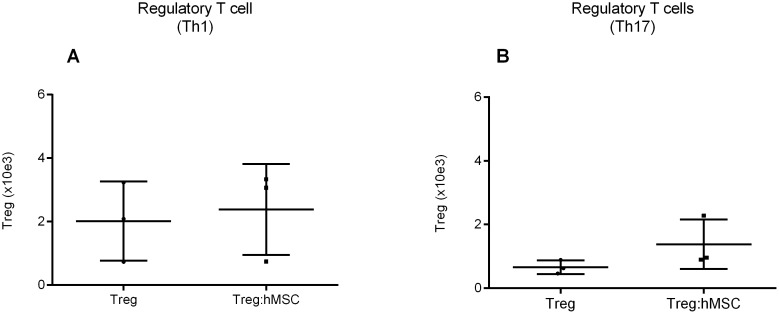
Presence of hMSCs do not switch the differentiation of naive T cells from IL-17A/IFN-γ secreting cells into regulatory T cells phenotype. (A) During Th1 differentiation, there is no significant differences between regulatory T cells numbers in presence of hMSCs (Treg:M) (2.38×10^3^±1.43×10^3^) and in absence (Treg) (2.02×10^3^±1.25×10^3^). (B) During Th17 differentiation, there is no significant differences between regulatory T cells numbers in presence of hMSCs (Treg:M) (0.66×10^3^±0.22×10^3^) and in absence (Treg) (1.38×10^3^±0.78×10^3^).

Since it has been previously described that hMSCs favor Treg differentiation instead of Th17 [36] we looked at the frequencies of Treg during differentiation to Th17 in the presence of hMSCs, but we did not find significant differences in the number of Treg as compared to the cultures without hMSCs (Figure 6). However, because of the observed lower frequencies of IFN-γ/IL-17- secreting cells the ratio between Treg and INF-y/IL-17A- secreting cells increased which would favor a regulatory environment. The FACS data are supplied in Figure S7.

## Discussion

**Figure 7:**
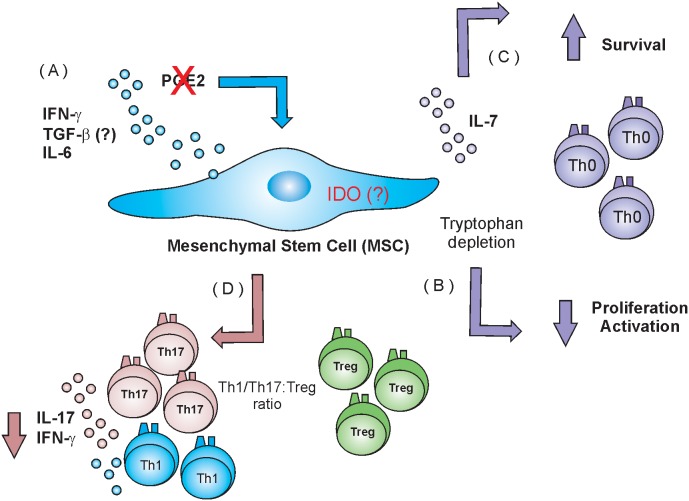
Schematic representation of hMSC interaction with lymphocytes. (A) Soluble molecule, but not prostaglandin E2, that will maybe activate and enhance IDO pathway on hMSCs (B) As IDO activation consequence there is tryptophan depletion from the environment leading to down regulation in lymphocytes proliferation (C) hMSCs IL-7 secretion will lead lymphocytes to survive without interfering with proliferation (D) For unknown reasons hMSCs will lead to a down regulation in INF-γ and IL-17 secretion by T lymphocytes altering the environmental ratio between Th1:IFN-γ, Th17:IL-17 secreting cells and T regulatory cells.

In this study we were able to identify some of the many activities exerted by hMSCs to control or down-regulate the immune response. Our main findings are related with an anti-proliferative, anti-apoptotic and anti-inflammatory effect of hMSCs on lymphocytes. We have confirmed the anti-proliferative effect and showed that hMSCs anti-apoptotic effect on lymphocytes, it is partially dependent of contact and related to IL-7. Another important finding it is that during Th1/Th17 differentiation *in vitro*, hMSCs presence have led to lymphocytes with reduced capacity of INF-γ and IL-17 secretion, regardless of having several pro-inflammatory cytokines in culture. Yet, due to these lower frequencies of IFN-γ/IL-17- secreting cells, the ratio changed between Treg and INF-γ/IL-17A-secreting cells increased which favors a regulatory environment. This changed ratio is very important to GvHD therapy [21], and links hMSCs to an anti-inflammatory role (Figure 7).

Our data showing that PHA and allogeneic stimulated lymphocyte proliferation it is suppressed by hMSCs independently of contact, agreed with those from Di Nicola and Krampera [10], [13]. In addition, Kim and Hemati have reported that hMSCs have the potential to alter macrophages phenotypes independently of contact with them [24].

Adding Indomethacin (IDT), a non-steroidal anti-inflammatory drug that inhibits cox-1 and 2 pathways, to the cultures revealed that the anti-proliferative effect of hMSCs on lymphocytes have occurred in a prostaglandin independent manner. Nemeth et al, has reported a prostaglandin effect on lymphocytes anti-proliferative responses hMSCs-driven in human sepsis via macrophages reprogramming. Our diverging results might be explained by absence of monocytes/macrophages in our cultures [25]. The fact that our cultures were monocyte-depleted allows us to suggest that the anti-proliferative effect observed is prostaglandin-independent. A number of studies indicate that IDO has anti-proliferative effects [12], [14], [26], [27], [28]. However, while some of these studies suggest that these effects are prostaglandin-dependent [12], some authors believe that not prostaglandin but other molecules such as IL-10, IFN-γ, TGF-β or IL-6 would be associated to these effects [26]–[29].

IDO acts by depleting the environmental available tryptophan, which may lead cells to apoptosis, though, this was not what we observed, which agrees with other studies [29]–[31]. Therefore, the mechanism that inhibited apoptosis in our co-cultures seems to be driven by IL-7. Cytokines such as IL-7, a member of the IL-2 family, has been reported as a possible molecule involved in apoptosis intrinsic and extrinsic pathways, by raising levels of anti-apoptotic proteins or inhibiting some pro-apoptotic proteins such as Bid, Bad or Bax [32]. IL-7 was described as a cytokine produced by several cells subsets such as T-cells, NK cells, monocytes and stromal cells [33], [34].

In fact, we found that IL-7 blockage in lymphocytes:hMSCs co-culture enhances significantly early apoptosis while addition of IL-7 to control cells impairs early and late apoptosis. This inhibition effect was similar to what was observed in hMSCs presence. Supporting our findings, IL-7 has been described as maintaining the cell viability by repressing cell death factors and/or activating a life factor [33], [35].

In addition, we observed that in co-culture the differentiation into Th1 and Th17 cells show a reduced capacity of INF-γ and IL-17 secretion, but there was not a significant increase in Treg differentiation in those co-cultures. Ghannam et al, also reported a reduced differentiation of Th17 in hMSCs presence, but to these authors, naïve cells were differentiating into a Treg phenotype instead to Th17 [36]. While we measured the amount of Treg cells considering the whole cell phenotype and FoxP3 protein expression, Ghannam et al measured FoxP3 by mRNA expression, which might explain why they obtained different results [36].

Taken together, our finds provide important preliminary results on the lymphocyte pathway modulated by hMSCs (Figure 7). Importantly we suggest that survival signals driven by IL-7 cytokine may play a prominent role on the apoptosis process. It remains unclear how IL-7 acts on CD3 T cells survival in our co-cultures and whether other molecules are involved with this process. Our findings pave the way to a better understanding on how hMSCs act on lymphocytes and may contribute for developing novel treatments and therapeutic targets for GvHD.

## Supporting Information

Figure S1 **Human mesenchymal stem cell (hMSC) phenotyping:** Isotype control showed in red, markers staining showed in blue. Cells were first gated on SSC vs FSC, than analyzed for each maker. Less than 1% of hMSCs expressed CD106, CD34, CD45, CD31, CD14 and HLA-DR. At least 93% of the analyzed hMSCs, expressed CD29, CD44, CD73, CD90 and CD105.
(TIF)

Figure S2 **hMSCs differentiation in 3 mesodermal lineages:** (A) Staining with Alizarin red the osteoblast differentiation control (B) Staining with Alizarin red the osteoblast differentiation, evidencing the calcium deposits (C) Staining with Toluidine blue the condroblast differentiation control (D) Staining with Toluidine blue the condroblast differentiation, evidencing the presence of proteoglycans (E) Staining with Oil red the adipocytes differentiation control (F) Staining with Oil red the adipocytes differentiation, evidencing the presence of intracellular lipid drops. (60x).
(TIF)

Figure S3 **Dendritic cells differentiation and maturation:** (A–E) CD14, CD209, CD80, CD83 and CD86 expression in M, iDC and mDC.
(TIF)

Figure S4 **PHA stimulated T lymphocytes proliferation.** (A) Gate on forward and side scatter (B) Gate selection of CD3 positive cells. (C) T lymphocytes without stimulus, control for KI-67 staining,. (D) PHA stimulated T lymphocytes proliferation (51.7%) in absence of hMSCs (E) PHA stimulated T lymphocytes proliferation (27.5%) in presence of hMSCs.
(TIF)

Figure S5 **PHA stimulated T lymphocytes apoptosis/necrosis.** (A) Control – T Lymphocytes stimulated with PHA stained only with Annexin-V (B) Control – T Lymphocytes stimulated with PHA stained only with propidium iodide (PI) (C) T Lymphocytes stimulated with PHA in absence of hMSCs, show late apoptosis/necrosis (39.5%) represented by cells that are double positive for PI/AnnexinV and the early apoptosis cells (31.2%%) represented by the single positive cell (Annexin-V). (D) Effect of hMSCs on lymphocytes apoptosis, result for late apoptosis/necrosis (15.9%) and the early apoptosis cells (14.3%).
(TIF)

Figure S6 **Graphic representation of gate strategy of lymphocytes cytokines production.** (A–B) Naive lymphocyte differentiation into Th1 in absence of hMSCs, gate on IFN-γ intracellular (38%) and in hMSCs presence, gate on IFN-γ intracellular (21%) (C–D) Naive lymphocyte differentiation into Th1 in absence of hMSCs, gate on IL-17A intracellular (6%) and in hMSCs presence, gate on IL-17A intracellular production (3%) (E–F) Naive lymphocyte differentiation into Th17, gate strategy of double positive cells for RORyt and IL-17A in absence of hMSCs (31.1%) and (16.6%) in hMSCs presence.
(TIF)

Figure S7 **Graphic representation of gate strategy of regulatory T cells.** In (A) Gate strategy of stimulated lymphocytes by scatter and CD45, (B) Gate on CD3 positive population (73%), (C) Gate strategy of double positive cells for CD3 and CD4 (55%), (D) Gate strategy in high CD25 (23.7%), (E) and low expression for CD127 (79.8%) and in (E) Gate strategy of double positive population for CD3 and FoxP3 expression (100%), (G–H) Fluorescence minus one (FMO) control for FoxP3 and CD25.
(TIF)
