# Supplementary figures and images for: Interleukin 7 Plays a Role in T Lymphocyte Apoptosis Inhibition Driven by Mesenchymal Stem Cell without Favoring Proliferation and Cytokines Secretion
Source: PLoS One. 2014 Sep 3;9(9):e106673. doi: 10.1371/journal.pone.0106673 (PMC4153662; doi:10.1371/journal.pone.0106673)

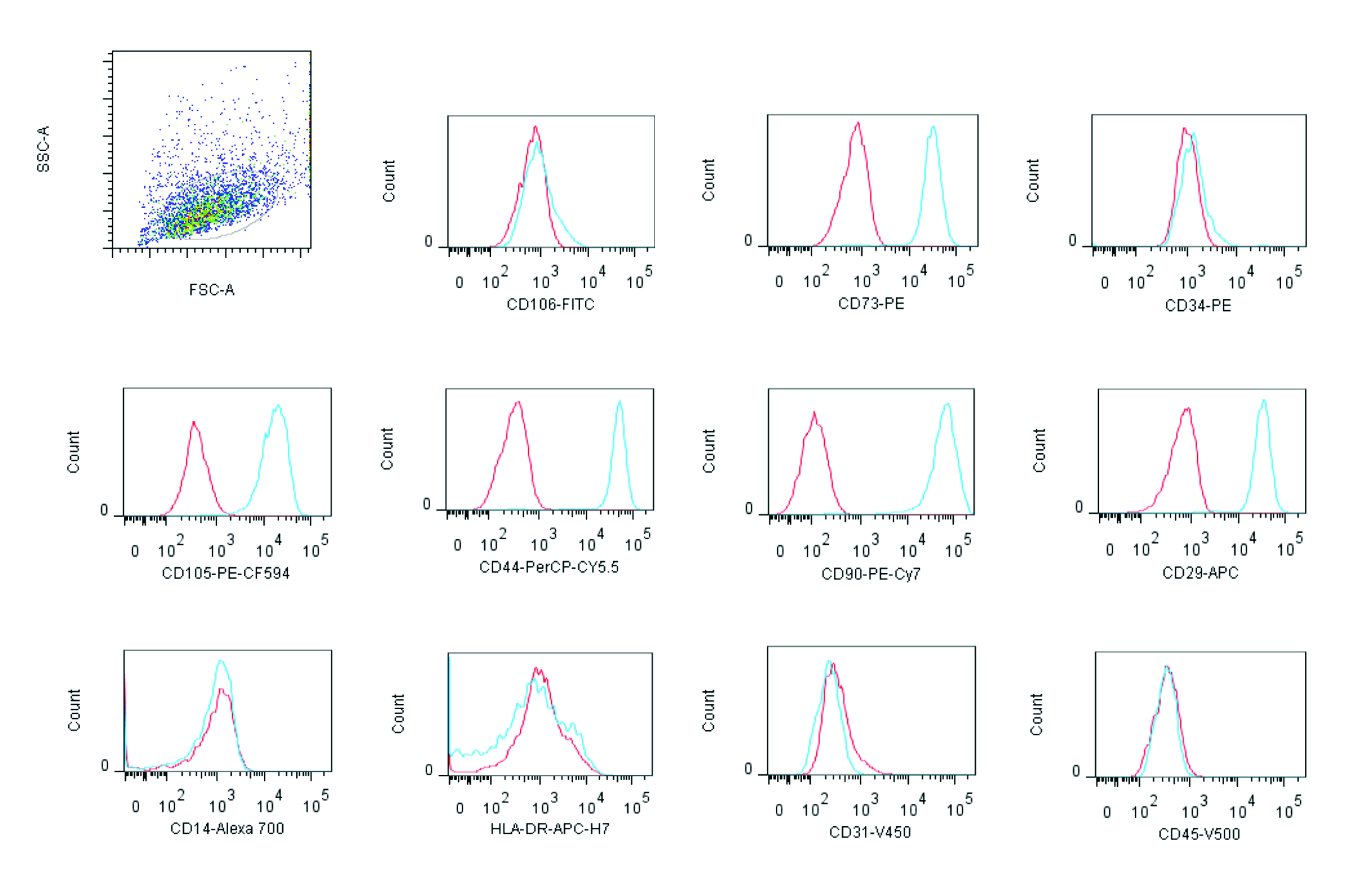

Supplement: Figure S1 — Human mesenchymal stem cell (hMSC) phenotyping: Isotype control showed in red, markers staining showed in blue. Cells were first gated on SSC vs FSC, than analyzed for each maker. Less than 1% of hMSCs expressed CD106, CD34, CD45, CD31, CD14 and HLA-DR. At least 93% of the analyzed hMSCs, expressed CD29, CD44, CD73, CD90 and CD105. (TIF) [file pone.0106673.s001.tif]

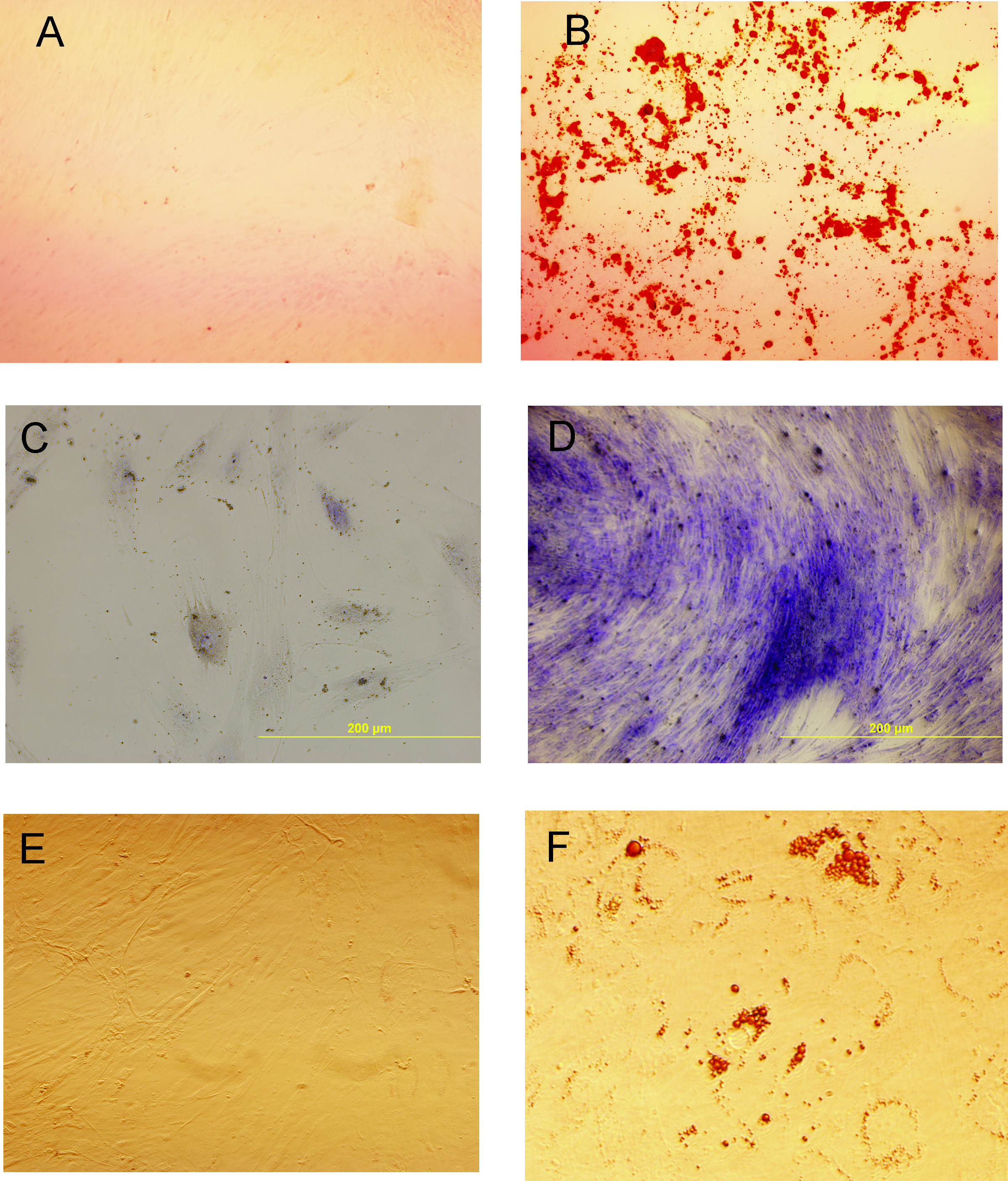

Supplement: Figure S2 — hMSCs differentiation in 3 mesodermal lineages: (A) Staining with Alizarin red the osteoblast differentiation control (B) Staining with Alizarin red the osteoblast differentiation, evidencing the calcium deposits (C) Staining with Toluidine blue the condroblast differentiation control (D) Staining with Toluidine blue the condroblast differentiation, evidencing the presence of proteoglycans (E) Staining with Oil red the adipocytes differentiation control (F) Staining with Oil red the adipocytes differentiation, evidencing the presence of intracellular lipid drops. (60x). (TIF) [file pone.0106673.s002.tif]

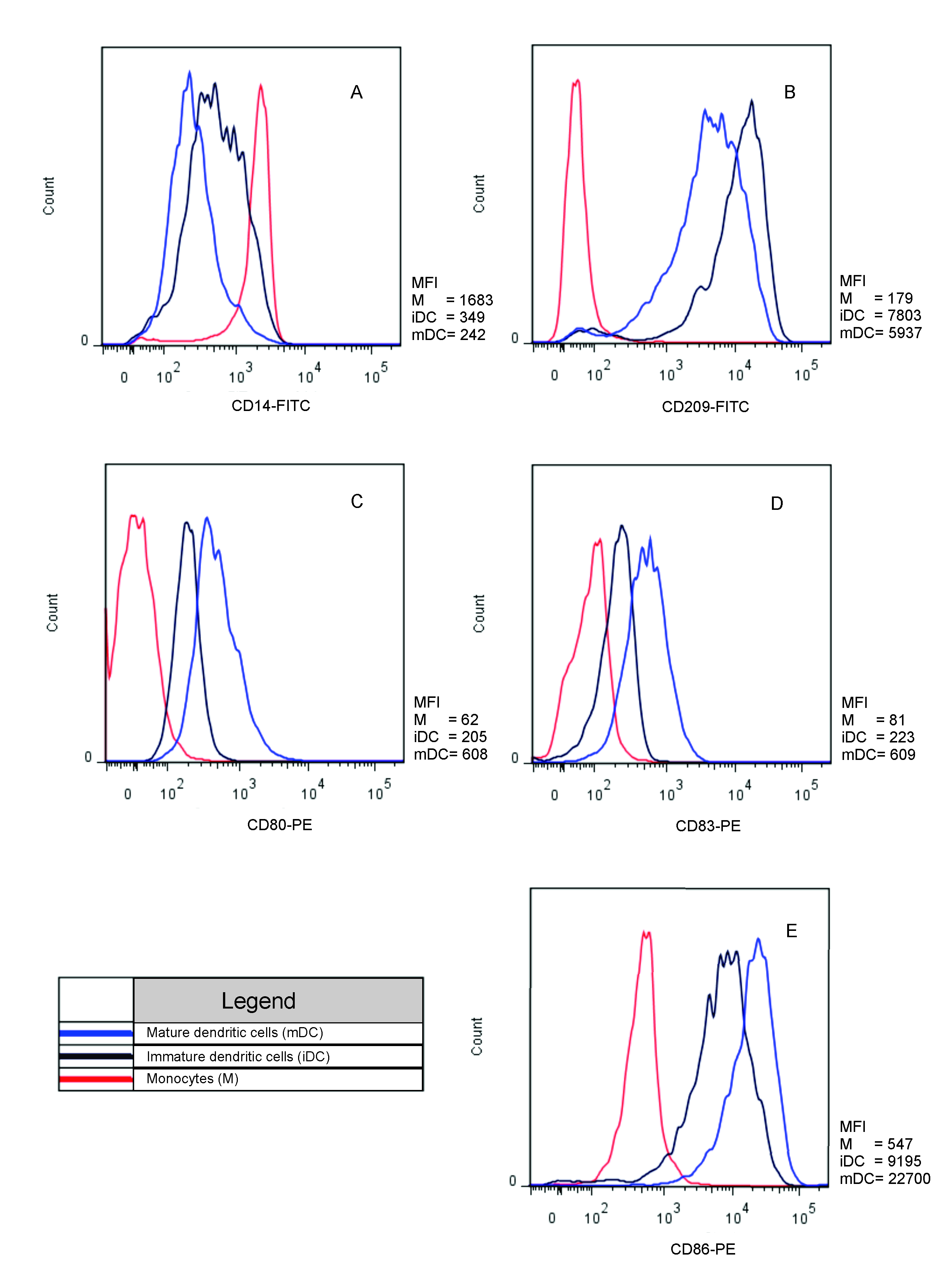

Supplement: Figure S3 — Dendritic cells differentiation and maturation: (A–E) CD14, CD209, CD80, CD83 and CD86 expression in M, iDC and mDC. (TIF) [file pone.0106673.s003.tif]

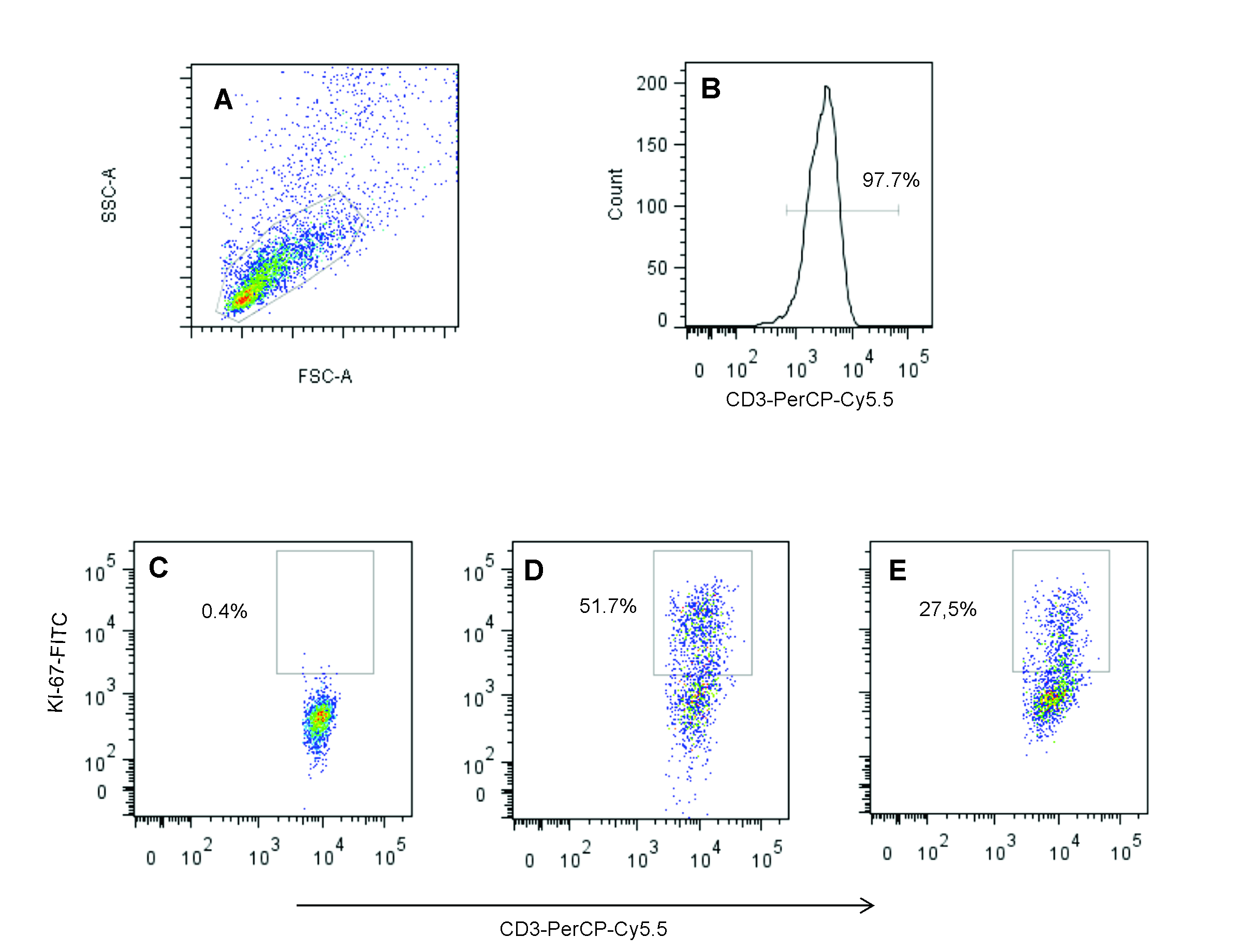

Supplement: Figure S4 — PHA stimulated T lymphocytes proliferation. (A) Gate on forward and side scatter (B) Gate selection of CD3 positive cells. (C) T lymphocytes without stimulus, control for KI-67 staining,. (D) PHA stimulated T lymphocytes proliferation (51.7%) in absence of hMSCs (E) PHA stimulated T lymphocytes proliferation (27.5%) in presence of hMSCs. (TIF) [file pone.0106673.s004.tif]

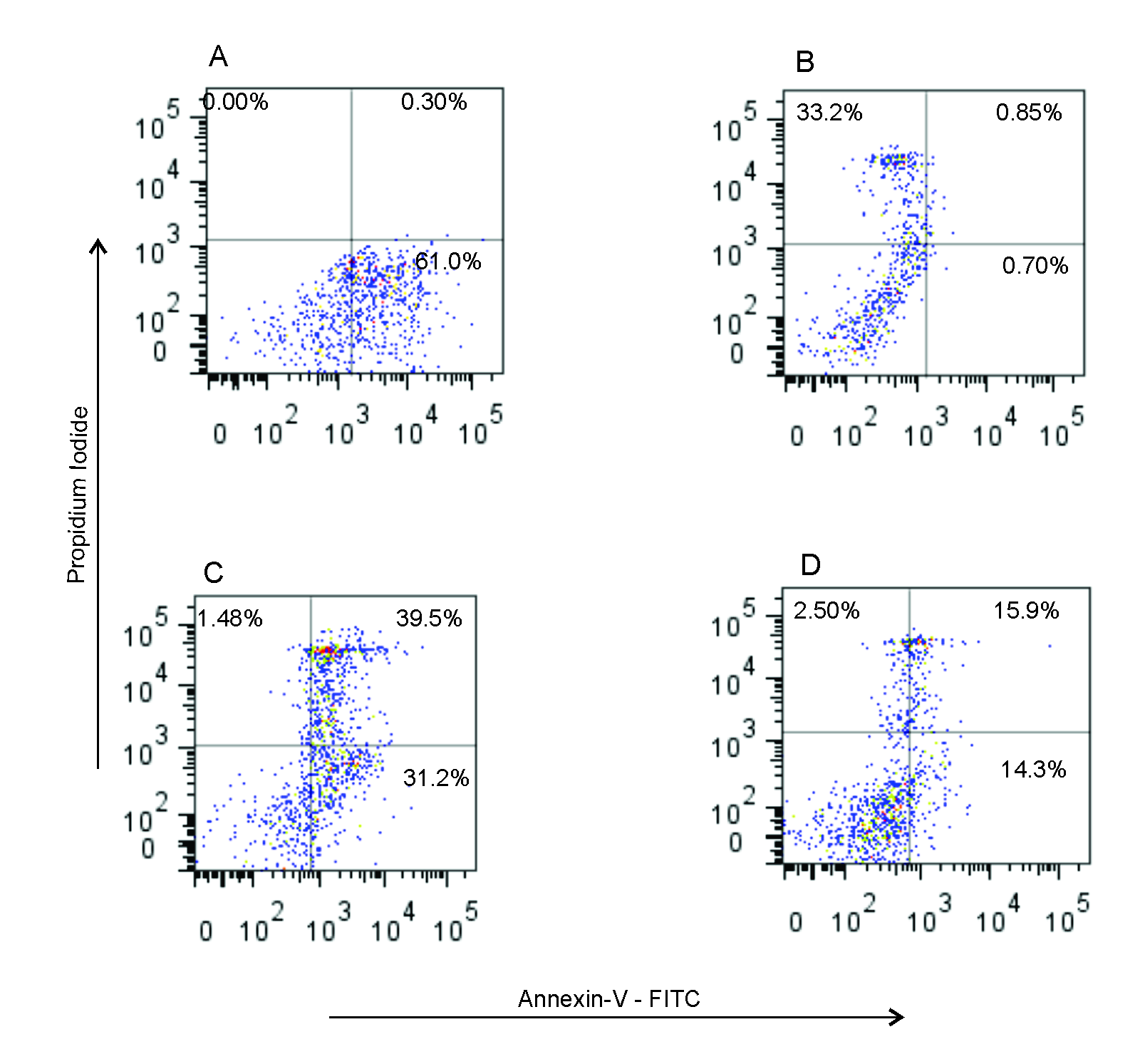

Supplement: Figure S5 — PHA stimulated T lymphocytes apoptosis/necrosis. (A) Control – T Lymphocytes stimulated with PHA stained only with Annexin-V (B) Control – T Lymphocytes stimulated with PHA stained only with propidium iodide (PI) (C) T Lymphocytes stimulated with PHA in absence of hMSCs, show late apoptosis/necrosis (39.5%) represented by cells that are double positive for PI/AnnexinV and the early apoptosis cells (31.2%%) represented by the single positive cell (Annexin-V). (D) Effect of hMSCs on lymphocytes apoptosis, result for late apoptosis/necrosis (15.9%) and the early apoptosis cells (14.3%). (TIF) [file pone.0106673.s005.tif]

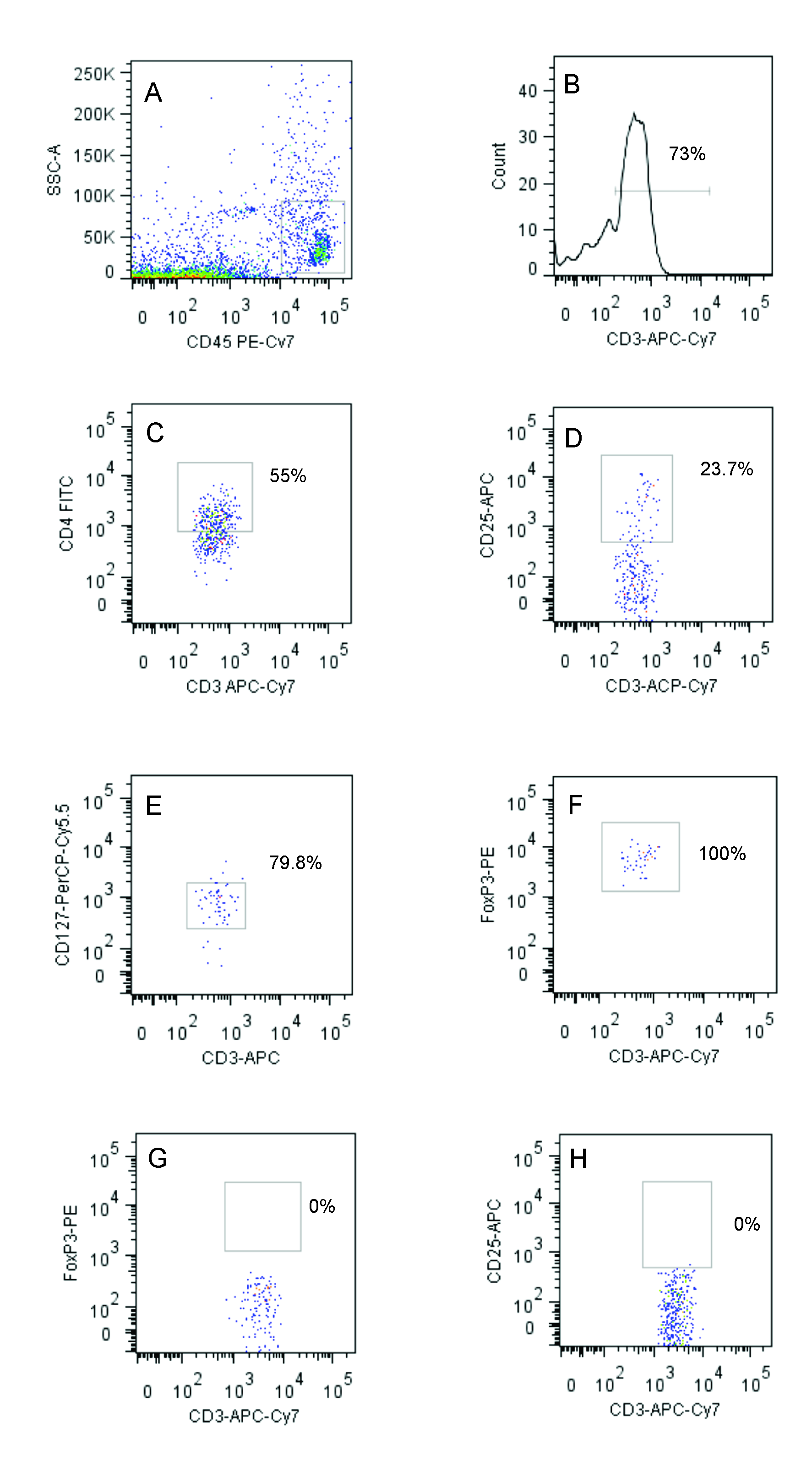

Supplement: Figure S7 — Graphic representation of gate strategy of regulatory T cells. In (A) Gate strategy of stimulated lymphocytes by scatter and CD45, (B) Gate on CD3 positive population (73%), (C) Gate strategy of double positive cells for CD3 and CD4 (55%), (D) Gate strategy in high CD25 (23.7%), (E) and low expression for CD127 (79.8%) and in (E) Gate strategy of double positive population for CD3 and FoxP3 expression (100%), (G–H) Fluorescence minus one (FMO) control for FoxP3 and CD25. (TIF) [file pone.0106673.s007.tif]
